# RNA Cytidine Acetyltransferase of Small-Subunit Ribosomal RNA: Identification of Acetylation Sites and the Responsible Acetyltransferase in Fission Yeast, *Schizosaccharomyces pombe*

**DOI:** 10.1371/journal.pone.0112156

**Published:** 2014-11-17

**Authors:** Masato Taoka, Daisuke Ishikawa, Yuko Nobe, Hideaki Ishikawa, Yoshio Yamauchi, Goro Terukina, Hiroshi Nakayama, Kouji Hirota, Nobuhiro Takahashi, Toshiaki Isobe

**Affiliations:** 1 Department of Chemistry, Graduate School of Science and Engineering, Tokyo Metropolitan University, Tokyo, Japan; 2 Core Research for Evolutional Science and Technology (CREST), Japan Science and Technology Agency (JST), Tokyo, Japan; 3 Department of Biotechnology, United Graduate School of Agriculture, Tokyo University of Agriculture and Technology, Tokyo, Japan; 4 Biomolecular Characterization Team, RIKEN Advanced Science Institute, Saitama, Japan; University of Cambridge, United Kingdom

## Abstract

The eukaryotic small-subunit (SSU) ribosomal RNA (rRNA) has two evolutionarily conserved acetylcytidines. However, the acetylation sites and the acetyltransferase responsible for the acetylation have not been identified. We performed a comprehensive MS-based analysis covering the entire sequence of the fission yeast, *Schizosaccharomyces pombe*, SSU rRNA and identified two acetylcytidines at positions 1297 and 1815 in the 3′ half of the rRNA. To identify the enzyme responsible for the cytidine acetylation, we searched for an *S. pombe* gene homologous to *TmcA*, a bacterial tRNA N-acetyltransferase, and found one potential candidate, *Nat10*. A temperature-sensitive strain of *Nat10* with a mutation in the Walker A type ATP-binding motif abolished the cytidine acetylation in SSU rRNA, and the wild-type *Nat10* supplemented to this strain recovered the acetylation, providing evidence that *Nat10* is necessary for acetylation of SSU rRNA. The *Nat10* mutant strain showed a slow-growth phenotype and was defective in forming the SSU rRNA from the precursor RNA, suggesting that cytidine acetylation is necessary for ribosome assembly.

## Introduction

The eukaryotic ribosome is a large ribonucleoprotein complex consisting of two major components, the small subunit and large ribosomal subunit. Each subunit is composed of one or more ribosomal RNA (rRNA) molecules and ∼80 proteins. The rRNAs form the basic ribosomal structure and a fundamental role in protein biosynthesis [1]–[5].

More than 30 types of post-transcriptional modifications (PTMs) have been identified at hundreds of rRNA sites in all three domains of life [6], [7]. Among the PTMs, acetylation at the N^4^ position of the pyrimidine ring of cytidine, resulting in N^4^-acetylcytidine (AcC), occurs in 5S, 16S, and 23S archaeal rRNA [8], [9] as well as in SSU rRNAs of a broad range of eukaryotes from budding yeast to mammals, while this acetylation does not occur in the bacterial rRNA [10]. The eukaryotic SSU rRNA has two potential AcCs [10], one of which is known to reside near the 3′-terminus [11]. For more than three decades, however, identification of the exact acetylation site has remained elusive [11], and little is known about its physiological significance in ribosome biogenesis and function or the presumptive acetyltransferase responsible for the modification.

Determination of RNA PTMs has typically depended on RNase mapping techniques, but the large molecular size of the eukaryotic SSU rRNA often precludes application of such techniques [6]. We recently developed an alternative method for the direct analysis of RNA using a nanoflow LC-coupled tandem MS (LC-MS/MS) technique coupled with the use of a DNA/RNA-based database, Ariadne, which allows for the unbiased identification and simultaneous chemical analysis of RNAs in complex biological mixtures [12]–[14]. With the use of Ariadne combined with genetics and molecular biology techniques, we were able to identify the positions of AcCs along the sequence of the SSU rRNA of the fission yeast, *Schizosaccharomyces pombe*, and we also determined the enzyme responsible for this modification. Based on our data, it appears that cytidine acetylation has a regulatory role in ribosome assembly, particularly in the process of SSU formation from a precursor rRNA.

## Experimental Procedures

### Chemicals

Standard laboratory chemicals were obtained from Wako Pure Chemical Industries (Osaka, Japan). RNase T1 was purchased from Worthington (Lakewood, NJ) and further purified by reverse-phase LC before use. Triethylammonium acetate buffer (pH 7.0) was purchased from Glen Research (Sterling, VA). The Dynamarker RNA High kit (oligonucleotide size markers) was obtained from BioDynamics Laboratory Inc. (Tokyo, Japan).

### Fission Yeast Strains, Genetic Methods, and Media

*S. pombe* strains including SP6 (a strain deficient in *leu1* gene function), ts447, and Nat10_G285D used in this study are listed in Table S1. *S. pombe* genetics procedures were carried out as described by Gutz *et al.* [15]. Complete medium (YE) (0.5% yeast extract, 2% glucose, 50 µg/ml adenine) and minimal medium (SD and EMM) [16] were used for the routine culture of *S. pombe*. The strains were constructed by mating haploids on sporulation medium [15] followed by tetrad dissection. Transformation of *S. pombe* cells was done using the lithium acetate method and the resulting transformant was selected by its auxotrophy [17].

To construct the diploid *Nat10* ^+/−^ strain, the 80-bp upstream and downstream regions of the *Nat10* ORF along with the 20 bp upstream and downstream of *ura4* were used as the primer sequences (Table S2). The gene *ura4* was amplified using the primers with pBluescript ura4^+^ plasmid [18] as a template. The resulting DNA carrying *Nat10* disrupted by *ura4* was used to transform the diploid *ura4^–/–^* strain. Stable *ura4* ^+^ transformants were selected, and proper replacement of the wild-type Nat10 allele with the disrupted construct was confirmed by PCR.

### Preparation of Cellular RNAs

Total RNA was prepared from *S. pombe* cells as reported [19]. Briefly, 2×10^8^ yeast cells were suspended in 0.3 ml of extraction buffer (0.5 M NaCl, 10 mM EDTA, 1% SDS, and 0.2 M Tris-HCl, pH 7.5) containing 0.3 ml phenol-chloroform and disrupted using a multi-beads shocker (Yasui Kikai, Osaka, Japan) in the presence of 0.5-mm-diameter glass beads (0.5 g). The supernatant was extracted again with 0.3 ml phenol-chloroform and then precipitated with the addition of ethanol.

rRNAs were purified by reversed-phase LC on a PLRP-S 4000 column (2×100 mm, 8 µm, Agilent Technologies, Santa Clara, CA, USA). After applying the total RNA (10 µg), the column was eluted with a 60-min linear gradient of 11.6–14% acetonitrile in 100 mM triethylammonium acetate (pH 7.0) containing 0.1 mM ammonium phosphate at a flow rate of 50 µl/min at 60°C while monitoring the eluate at 260 nm [20]. rRNA with >95% purity was used for this study.

### Sequence-specific RNase H Cleavage of rRNAs

rRNA (2 pmol) was digested with 15 U of RNase H (Takara Bio, Shiga, Japan) at 42°C for 1 h, guided by a synthetic 20–28-mer of DNA (4 pmol) complementary to the cleavage site in 100 µl volume of 40 mM Tris–HCl (pH 7.7) containing 4 mM MgCl_2_ and 1 mM DTT [21]. All DNA sequences used for the cleavage are presented in Table S2. Before adding the enzyme, the sample was denatured at 65°C for 10 min. The reaction was stopped by addition of 8 µl of 0.5 M EDTA, and the resulting RNase H fragments were separated on a PLRP-S column 300 column (2 mm i.d. ×100 mm, 3 µm particles, Agilent Technologies) with a 60-min linear gradient of 13.6–16.4% acetonitrile in 100 mM triethylammonium acetate (pH 7.0) containing 0.1 mM ammonium phosphate at a flow rate of 50 µl/min at 60°C.

### Construction of cDNAs and Plasmids

Sequence homology of the N-acetyltransferase domain in TmcA (residues 356–531, accession number, P76562) was searched by BLAST program against the database of open reading frame of *S. pombe*. To obtain the cDNA homologue, The cDNA library was prepared using the PrimeScript RT-PCR kit (Takara Bio, Ohtsu, Japan). A 3-kb fragment carrying the *Nat10* ORF was amplified by PCR using the primers listed in Table S2. The product was cloned into the site between BamHI-XhoI of the multi-copy expression vector pREP1 containing the *nmt* promoter and *leu2* [22] using the In-Fusion HD Cloning kit (Takara Bio, Shiga, Japan). The resulting plasmids from SP6 *S. pombe* and Nat10_G285D were designated as pREP1-Nat10_wt and pREP1-Nat10_G285D, respectively.

### Nanoflow LC-MS Apparatus for RNA Analysis

The LC system used was essentially as described [14], consisting of a nanoflow pump (LC Assist, Tokyo, Japan) and a ReNCon gradient device. The column was prepared with a fused-silica capillary (150 µm i.d.×375 µm o.d. ×120 mm length) packed with a reversed-phase material (Develosil C30-UG-3, particle size 3 µm, Nomura Chemical, Aichi, Japan). LC was performed at a flow rate of 100 nl/min using a 60-min linear gradient of 10–32% acetonitrile in 10 mM triethylammonium acetate (pH 7.0). The eluate was introduced into an LTQ-Orbitrap hybrid mass spectrometer (Thermo Fisher Scientific, San Jose, CA, USA) through an electrospray ion source operating in negative mode at −1.4 kV. The mass spectrometer automatically switched between Orbitrap-MS and linear ion trap–MS/MS acquisition modes [14]. Survey full scan mass spectra (from m/z 500 to 1950) were acquired at a mass resolution of 100,000. Up to 4 most intense mass peaks that exceeded the intensity of 10,000 counts/sec were isolated with a 3 m/z window for fragmentation. Precursors were fragmented by colision induced dissociation in iontrap with a normalized collision energy of 25%.

### Database Search and Interpretation of MS/MS RNA Spectra

Ariadne [12] was used for database searches and assignment of MS/MS RNA spectra. We used the genome database of *S. pombe* as a resource in conjunction with Ariadne (http://www.pombase.org/downloads/datasets). The following search parameters for Ariadne were used: the maximum number of missed cleavages was set at 1; the variable modification parameters were one methylation per RNA fragment for any residue; RNA mass tolerance of ±20 ppm and MS/MS tolerances of ±750 ppm were allowed.

### Sucrose Density Gradient Fractionation

Fractionation was performed as described [23], [24]. Briefly, cells were grown logarithmically to 1×10^7^ cell/ml, and then cycloheximide was added to a final concentration of 0.1 mg/ml; the culture was then chilled on ice for 10 min. The cells were then precipitated by centrifugation and resuspended in 4% of the original culture volume of 20 mM Tris-HCl (pH 7.5), 140 mM KCl, 5 mM MgCl_2_, 0.5 mM DTT, 1% (w/v) Triton X-100, 0.1 mg/ml cycloheximide, and 0.2 mg/ml heparin. After washing, the cells were resuspended in 1% of the original culture volume of the same buffer and disrupted by using the Yasui Kikai multi-beads shocker in the presence of 0.5-mm-diameter glass beads (0.5 g/20 ml cell culture). The lysate was cleared by centrifuging twice and then stored at −80°C. Polysomes were separated by loading the lysate onto a 15–50% (wt/vol) gradient of sucrose followed by sedimentation ultracentrifugation in a Beckman MLS50 rotor for 2.5 h at 45,000 rpm at 4°C. Each sucrose gradient was fractionated using isotonic pumping of 60% sucrose from the bottom and monitoring of the eluent at 254 nm.

### Other Procedures for RNA Analysis

Agarose gel electrophoresis and in-solution digestion of RNA were performed as described [25]. Tertiary structure of RNA defined by the PDB file was analyzed with Swiss-Pdb viewer (http://spdbv.vital-it.ch/).

## Results

### Determination of Acetylcytidine Positions of SSU rRNA

To identify the acetylation sites on eukaryotic SSU rRNA, we purified 18S rRNA from *S. pombe*, digested the RNA with RNase T1, and analyzed the fragments using a nanoflow LC-MS/MS system. Using Ariadne software to analyze the resulting MS data, we assigned all RNase T1 fragments covering the sequence region of the 1842-bp eukaryotic SSU rRNA, excluding the free guanosine monophosphates (Fig. S1). In fission yeast SSU rRNA, we found 26 fragments carrying a modified nucleotide resulting from PTM.

The modifications included two individual AcCs, one in each of the sequences UUUC(AcC)Gp and C(AcC)Gp (Table S3). One of the fragments with the sequence UUUCCGp was unique to the fission yeast SSU rRNA sequence and assigned to the position ^1811^UUUC(AcC)^1816^Gp. The other sequence, CCGp, appeared nine times in the fission yeast SSU rRNA at positions 285–287, 575–577, 664–666, 866–868, 1048–1050, 1296–1298, 1476–1478, 1541–1543, and 1758–1760 (Table S3). To discriminate between redundant sequences, we prepared seven large fragments of rRNA (1–170, 183–538, 554–693, 703–986, 1003–1162, 1177–1428, and 1445–1842) using limited RNase H cleavage, purified them by reversed-phase LC, and digested each with RNase T1. Data from the subsequent nanoflow LC-MS/MS analysis showed only C(AcC)Gp in the 1177–1428 RNase H fragment, which allowed the identification of the AcC at ^1296^C(AcC)^1298^Gp (Fig. S2). Thus, the results clearly showed that the fission yeast SSU rRNA has two AcCs at positions 1297 and 1815.

### The N-acetyltransferase Encoded by *Nat10* Is Responsible for the Cytidine Acetylation of Eukaryotic SSU rRNA

To identify the gene responsible for the cytidine acetylation of SSU rRNA, we searched the *S. pombe* protein database (http://www.pombase.org/) for a protein having an amino acid sequence similar to that of the N-acetyltransferase domain of bacterial tRNA acetylation enzyme *TmcA* [26]. The search identified a single gene, *Nat10* (systematic name, SPAC20G8.09c) (Fig. S3). The predicted protein product of *Nat10* (Nat10p) has a domain structure similar to that of the *TmcA* product; the four conserved domains within *Nat10* are DUF1726, helicase_RecD, GNAT_acetyltr_2, and tRNA_bind_2 (given as Pfam ID). In addition to the structural similarity, Nat10p appeared to be a strong candidate cytidine N-acetyltransferase for the fission yeast] SSU rRNA because (i) Nat10p localizes in nucleolus [27], where rRNA is transcribed and assembled into ribosomes, and (ii) a repressible mutant of the gene *KRE33*, a *Nat10* ortholog in *Saccharomyces cerevisiae*, has a defect in the polysome profile during the biogenesis of the small ribosomal subunit and causes an accumulation of 35S pre-rRNA [28].

**Figure 1:**
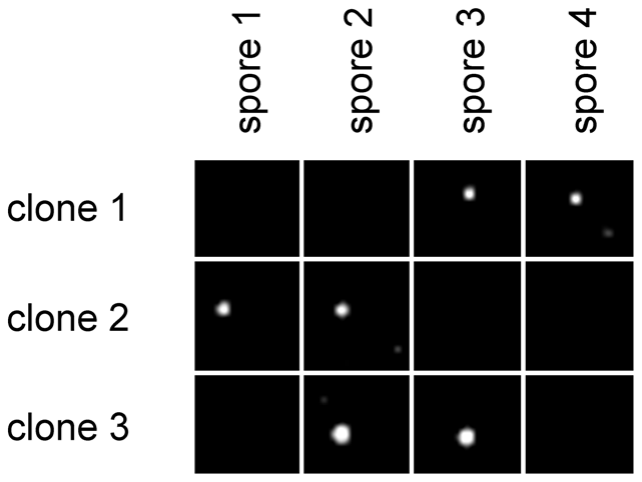
The *Nat10* null mutant is inviable. Tetrad dissection experiments were carried out using segregates shown below the panel. The four spores from each given tetrad are grouped horizontally. The dissected spores were grown at 30°C for 3 days on YE medium. Parental strain: *h+/h– leu1-32/+ his3-D1/+ ura4-D18/ura4-D18 nat10::ura4/+*.

**Figure 2:**
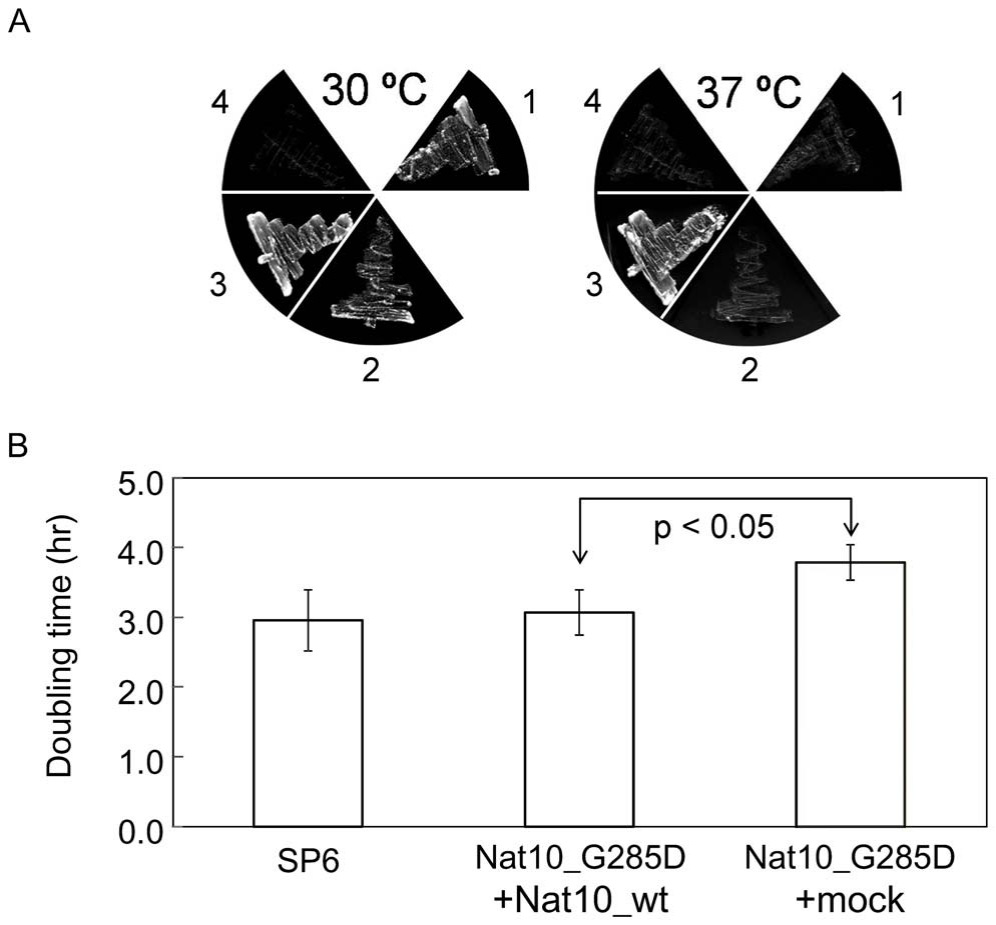
Analysis of the Nat10_G285D temperature-sensitive strain. A. Effects of *Nat10* on the temperature sensitivity of the Nat10_G285D mutant. The Nat10_G285D strain (deficient in *leu1* gene) was supplemented with *Nat10* cDNA in pREP1 vector containing the nmt1 promoter and an auxotrophic marker, *leu2* gene, and incubated for 3 days on an SD plate without leucine at 30°C or 37°C. The cDNAs used were: 1) mock vector; 2) Nat10_G285D cDNA in the vector; 3) wild-type Nat10 cDNA in the vector; and 4) no vector and cDNA. Note that the temperature-sensitive mutant grows at 37°C with the expression of *Nat10* cDNA. B. Effects of *Nat10* on the growth rate of Nat10_G285D mutant. Doubling time during the logarithmic growth phase (5.0×10^6^ to 2.5×10^7^ cells/ml) at 30°C was measured in SD medium without leucine. The strains used were Nat10_G285D supplemented with pREP1-Nat10_wt or the mock vector. Each value represents the mean ± standard deviation of three independent assays. The arrow indicates a significant difference as determined by the Student's t test (p<0.05). Note that the growth rate of the Nat10_G285D mutant was recovered upon expression of *Nat10* cDNA.

We utilized genetics to examine whether the mutation of Nat10p affects the phenotype of *S. pombe*. Our attempts to construct a *Nat10* knockout strain in haploid yeast were unsuccessful. Instead, we prepared a *Nat10* null strain from diploid yeast using tetrad dissection [15], but the resulting strain was inviable (Fig. 1). Thus, we utilized a *Nat10* temperature-sensitive mutant designated previously as “strain 447,” which carries a mutation in *Nat10* and has a cell-cycle progression defect [29]. We confirmed that this strain was in fact temperature sensitive and that this sensitivity was rescued by complementation with wild-type, but not by mutant, *Nat10* cDNA (Fig. 2A). This mutant strain had a doubling time of 200-min in YE medium at 30°C, which was about 1.9 times slower than that of wild-type yeast; however, the growth rate of this mutant was also recovered to the wild-type levels by ectopic expression of wild-type *Nat10* cDNA (Fig. 2B). These results suggested that the mutation in *Nat10* was responsible for the temperature sensitivity and the slow-growth phenotype.

**Figure 3:**
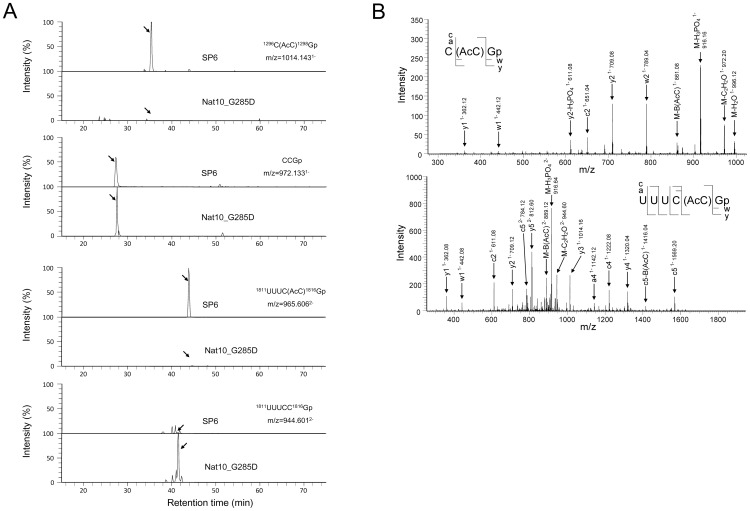
MS-based identification of acetylcytidines in *S. pombe* 18S rRNA. A. Extracted ion monitoring of RNase T1 fragments of 18S rRNA containing acetylcytidine (AcC) and cytidine (C)-1297 (upper panel) and AcC and C-1815 (lower panel). The 18S rRNAs were purified from strain SP6 or Nat10_G285D grown at 30°C in YE medium, digested by RNase T1 and applied to the LC-MS system (50 fmol each). The sequences and m/z values of AcC and C-containing nucleotides are indicated. A mass window of 3 ppm was used for the extractions. Y axis indicates the peak intensity relative to the most intensive peak in each panel. Note that the MS signals of AcC-containing nucleotides were completely lost in the Nat10_G285D mutant strain (indicated by arrows). B. MS/MS spectra of AcC-containing fragments. The acetylated RNA ions [C(AcC)Gp^1−^, m/z 1014.14; UUUC(AcC)Gp^2−^, m/z 965.60, in A) were analyzed by collision-induced dissociation. The position of acetylcytidine residues was identified by manual interpretation of the a-, c-, w- and y-type series ions and other specific product ions as indicated in the figure. The series ions assigned are indicated on the RNA sequence in the inset.

**Figure 4:**
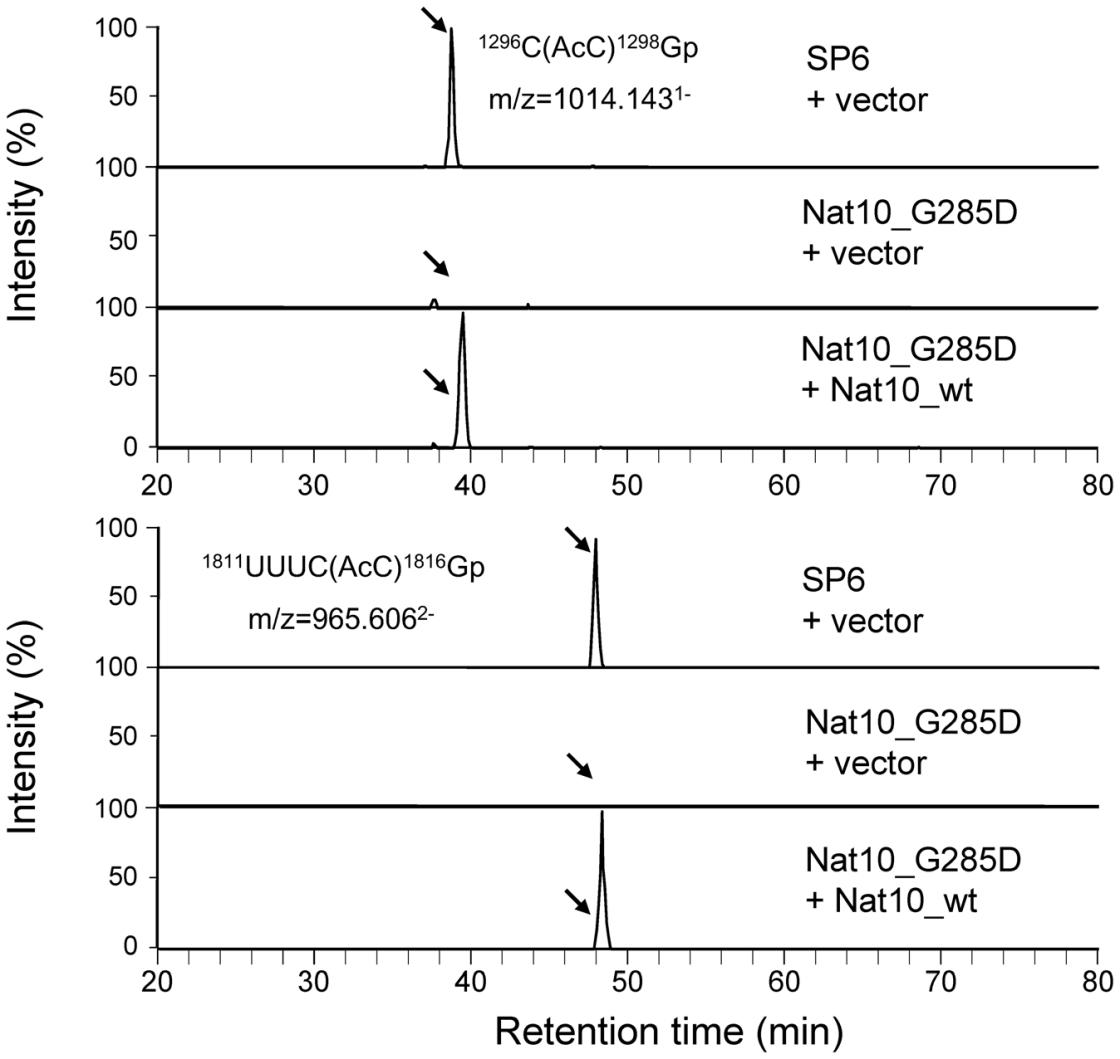
Effect of *Nat10* on the acetylation of cytidines 1297 and 1815 in 18S rRNA. Extracted ion monitoring of RNase T1 fragments of 18S rRNA containing AcC-1297 (upper panel) and AcC-1815 (lower panel) is shown. The analysis was performed for 18S rRNAs purified from strain SP6 supplemented with the mock vector, strain Nat10_G285D supplemented with the mock vector, or strain Nat10_G285D supplemented with pREP1-Nat10_wt, respectively, as indicated. Each yeast strain was grown at 30°C in EMMmedium without leucine. The rRNA was digested by RNase T1 and applied to the LC-MS system (50 fmol each). The sequences and m/z values of AcC-containing nucleotides are indicated. A mass window of 3 ppm was used for the extractions. Y axis indicates the peak intensity relative to the most intensive peak in each panel. Note that the MS signals of AcC-containing nucleotides at the positions indicated by arrows appear in the Nat10_G285D mutant strain upon expression of *Nat10* cDNA.

Sequence analysis showed that strain 447 has a point-mutation in *Nat10* that results in a single amino acid substitution (Gly to Asp) at position 285; we thus renamed the strain Nat10_G285D. Gly-285 is conserved among Walker A motifs of the *TmcA* family of N-acetyltransferases in the consensus sequence AxR*GRGKT/S (*G indicates the position of Gly-285) and acts as a site of interaction with the phosphate group of ATP in the helicase domain [30]. The LC-MS/MS analysis of SSU rRNA obtained from the strain Nat10_G285D grown at the permissive temperature (30°C) revealed that the mutant SSU rRNA lacks the cytidine acetyl groups at positions 1297 and 1815 (Fig. 3). We also found that ectopic expression of *Nat10* in this mutant recovered the acetylation of both cytidines almost completely to the wild type level as estimated form the peak intensity in Fig. 4, demonstrating that Nat10p is responsible for the cytidine acetylation of SSU rRNA.

### Lack of SSU rRNA Acetylation Affects Ribosome Assembly

**Figure 5:**
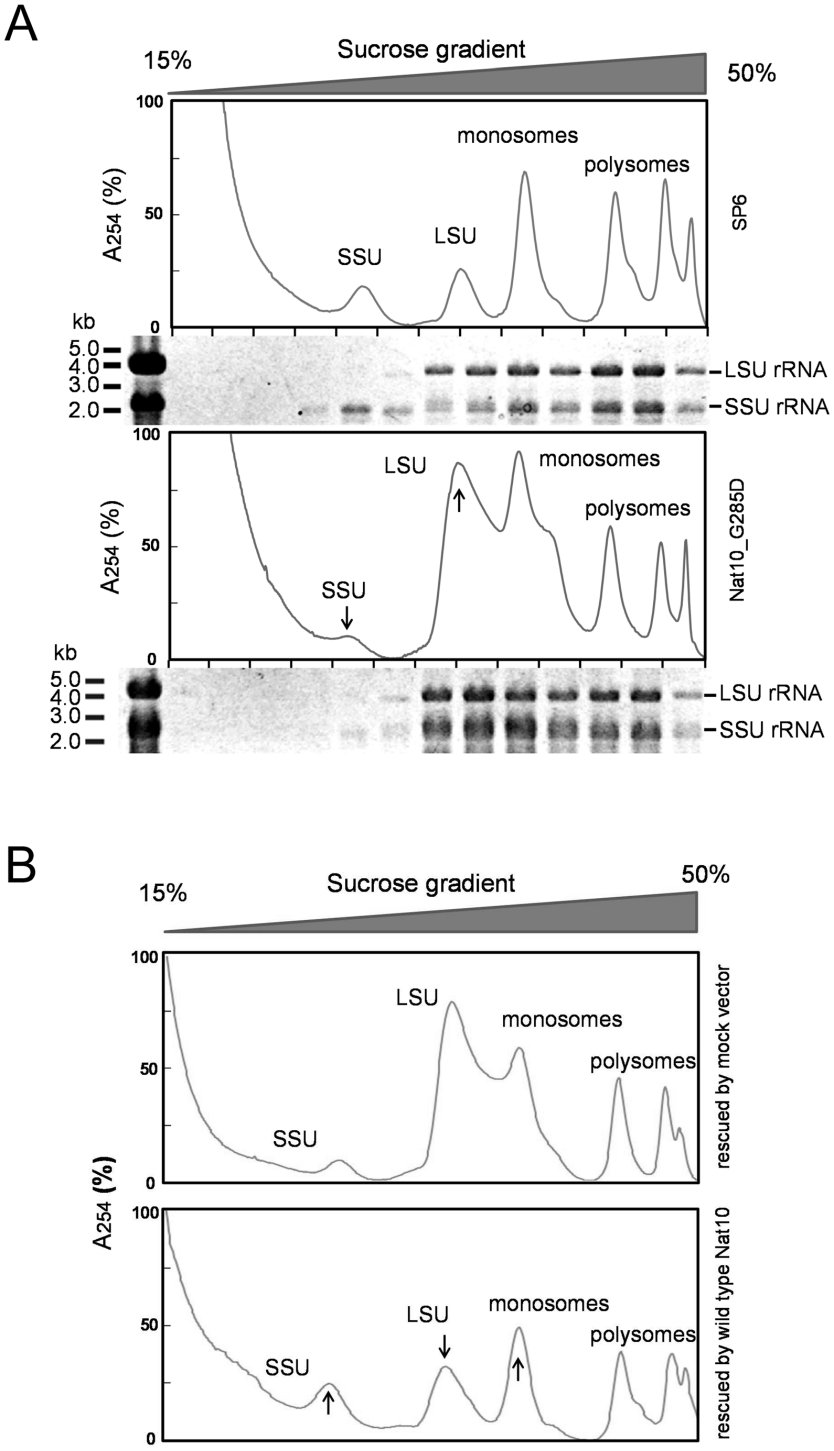
Effect of *Nat10* on ribosome assembly. A. Ultracentrifugation analysis of ribosome assembly in the Nat10_G285D mutant. Ribosomal and polysomal fractions prepared from strain SP6 (upper panel) or Nat10_G285D mutant (lower panel), grown at 30°C in YE medium were analyzed with 15–50% sucrose density gradient centrifugation. The UV profiles (A_254_) of the separation are depicted. The scales under each UV profile denote individual fractions, and the electrophoretograms shown below the UV profile are the results of agarose gel electrophoresis of each fraction. B. Effect of *Nat10* on ribosome assembly of the Nat10_G285D mutant. Ribosomal and polysomal fractions derived from strain Nat10_G285D supplemented with the mock vector (upper panel) or with pREP1-Nat10_wt (lower panel) grown at 30°C in EMM medium without leucine were analyzed with 15–50% sucrose density gradient centrifugation under the conditions as in A. Note that the apparently aberrant phenotype in ribosome assembly of the Nat10_G285D mutant was rescued upon expression of *Nat10*, as indicated by the decrease in free large subunit (LSU) and increase in SSU and monosomes.

To examine whether the observed slow growth of strain Nat10_G285D might be caused by defects in ribosomal assembly or function, we prepared a crude cellular extract from this mutant strain and analyzed the ribosomal and polysomal fractions by sucrose density gradient centrifugation. In the mutant strain, the free large subunit complexes and 80S monosomes accumulated to abnormal levels compared with the wild-type strain, with a concomitant decrease in the proportion of SSU complexes (Fig. 5A). Agarose gel electrophoresis–based analysis of the rRNAs in each fraction showed that the mutant strain contained only a trace amount of free SSU complex and large amounts of aberrant complexes containing SSU rRNAs with a relatively broad range of sedimentation than the normal complexes found in the wild-type strain. This abnormal repertoire of assembled ribosomes was rescued almost completely by ectopic expression of wild-type *Nat10* cDNA in the mutant strain (Fig. 5B). Because strain Nat10_G285D lacks cytidine N-acetyltransferase activity, cytidine acetylation of SSU rRNA may have a role in the maturation of precursor rRNAs and the ability to generate SSU complexes during ribosome assembly.

## Discussion

We report the sites of acetylation in the fission yeast SSU rRNA and the corresponding N-acetyltransferase responsible for this acetylation. A temperature-sensitive strain of *Nat10* abolished the acetylation of SSU rRNA and exhibited aberrant ribosome formation. Because the supplemented wild-type *Nat10* cDNA recovered the acetylation and rescued the slow-growth phenotype, the cytidine acetylation appears to be essential for normal ribosome assembly *via* the SSU rRNA formation. We identified two acetylcytidines in the fission yeast SSU rRNA at positions 1297 and 1815; however, whether both of those acetylcytidines are required for the observed phenotype of yeast cells remains to be determined.

The nucleotide sequences around the two acetylcytidines at positions 1297 and 1815 are extremely well conserved among SSU rRNAs of a broad range of eukaryotes, including yeast, slime molds, rats, and humans (Fig. S4). In the tertiary structure of SSU rRNA, cytidines 1297 and 1815 are located in helix 34 and 45, respectively (Fig. S5), which exist near the mRNA decoding center of the ribosome [31], [32]. Within the rRNA structure, cytidines 1297 and 1815 appear to stabilize helices 34 and 45 via a hydrogen bond to guanosines 1448 and 1831, respectively (3U5B, RCSB Protein Data Bank, http://www.rcsb.org/pdb/home/home.do). It should be noted, however, that the currently available tertiary structure of SSU ribosome does not incorporate PTM information in rRNA [32]. Because of the impact of PTMs on RNA structure, we suggest that the current structural model of the SSU ribosome be reconstructed so as to incorporate the cytidine acetylation data reported here. Based on our findings, the acetyl group at N4 of the cytidine pyrimidine ring should prevent the formation of a hydrogen bond to the carbonyl group at C6 of the purine ring of guanosine, thereby altering the backbone structure of helices 34 and 45 as seen in the current structural model of SSU ribosome (Fig. S5).

The protein product encoded by *TmcA* has an N-acetyltransferase domain that utilizes acetyl-CoA as an acetyl group donor [26] and an ATP hydrolysis–driven RNA helicase domain that delivers the modification site to the active center of the N-acetyltransferase domain [30]. Because Nat10p has a similar amino acid sequence to *TmcA* N-acetyltransferase (24% identity) and similar domain structure, we speculate that Nat10p and *TmcA* have similar catalytic mechanisms; however, this requires experimental evidence. Presumably, Nat10_G285D N-acetyltransferase lost its activity within SSU rRNA because the point mutation at Gly-285 in the Walker A motif of its RNA helicase domain caused a defect in ATP hydrolysis necessary for the subsequent cytidine acetylation reaction (Fig. 3A). We also noted that the structure and function of the N-acetyltransferase encoded by *Nat10* has been conserved throughout evolution, because the human ortholog of *Nat10* (accession number, Q9H0A0) has a very similar encoded amino acid sequence to its yeast counterpart (Table S4) and thus could replace the yeast enzyme in the cytidine acetylation of the fission yeast SSU rRNA (data not shown).

Strain Nat10_G285D showed a slow-growth phenotype but was viable despite the absence of two acetylcytidines in the SSU rRNA. However, we found that the *Nat10* null strain was inviable, indicating that cell viability was independent of SSU rRNA acetylation. Yeast Nat10p shows >47% sequence similarity with eukaryotic Nat10ps from various species (Table S4). It has been reported that downregulation of the *Nat10* ortholog of the nematode *Caenorhabditis elegans* perturbs expression patterns of sex-specific gonadal markers [33] and its mutation in the putative N-acetyltransferase domain affects key traits such as brood size, age at sexual maturity, sperm number, and rate of progeny production [34]. Likewise, the human *Nat10* ortholog is thought to play a role in regulating cytokinesis, mitotic chromosome decondensation, and telomerase expression, probably owing to its ability to transfer the acetyl group to both histones and α-tubulin [35]–[38]. More recently, Larrieu *et al.* reported that human *Nat10* affects lamin A/C–mediated nuclear architecture presumably owing to its affects on α-tubulin and the altered chromatin organization associated with cancer and laminopathies, including the premature-aging disease Hutchinson-Gilford progeria syndrome [39]. Thus, the N-acetyltransferase encoded by *Nat10* participates in a variety of biological processes owing to its broad range of target substrates, including rRNA reported here and subsets of proteins such as histones. The *Nat10* null strain proved inviable even though the yeast mutant that lacked the acetylcytidines in SSU rRNA was viable, indicating that further studies are needed to elucidate the *Nat10* N-acetyltransferase function.

*Note added in proof*: After submission of this manuscript, a paper appeared reporting that budding yeast homolog of Nat10p acetylates cytidine-1773 in the 18S rRNA of *Saccharomyces cerevisiae* and modulates the processing of the 23S precursor to yield 18S rRNA [40]. This cytidine residue corresponds to one of the cytidines, *i.e.*, cytidine-1815, identified as the substrate of *S. pombe* Nat10p reported in this study.

## Supporting Information

Figure S1 Nucleotide sequence of *S. pombe* 18S rRNA with the sites of modification identified by the LC-MS/MS. Black solid bars denote RNase T1–digested 18S rRNA fragments identified by this analysis. When the bar continues to the next line, a dotted line is added to the bar. The sequences with or without modifications were identified by Ariadne and confirmed by manual inspection of the MS/MS spectra. Each N-acetylcytidine is indicated by a red arrowhead. The fragment carrying modified residues is noted in Table S3. Abbreviations of modified residues (blue letters) are as follows: 2′-O-methyladenosine; B, 2′-O-methylcytidine, #, 2′-O-methylguanosine; J, 2′-O-methyluridine; M, N^4^-acetylcytidine; α, 1-methyl-3-(3-amino-3-carboxypropyl) pseudouridine; 7, 7-methylguanosine; ζ, N^6^,N^6^-dimethyladenosine.
(TIFF)

Figure S2 Site of acetylation of CCG in an 18S rRNA fragment as determined after digestion with RNase H. Extracted ion monitoring is indicated for each of CCGp and C(AcC)Gp obtained from an RNase T1 digest of each RNase H fragment. The extracted mass of each ion was: (upper chromatogram) [CCGp]^−^, m/z = 972.133 and (lower chromatogram) [C(AcC)Gp]^−^, m/z = 1014.143. Mass windows of 5 ppm were used for extraction. Y axis expressed the most intense peak as 100% among the set of extracted ion chromatograms when 50 fmol of purified 18S rRNA was applied to the system. Residues of the RNase H fragment (bold) and candidate CCG positions (parentheses) are indicated on the left. Note that the C(AcC)Gp fragment was detected only in RNase H fragment 1177–1428. The oligonucleotide sequence containing C(AcC)G was confirmed by MS/MS analysis (data not shown).
(TIFF)

Figure S3 Structural similarity of *Escherichia coli TmcA* and *S. pombe Nat10*. (A) The N-acetyltransferase domain of *TmcA* was used to search for a homolog in *S. pombe* (http://www.pombase.org/). The domain shows sequence similarity with the acetyltransferase domain of *Nat10*. Amino acid identity: strong similarity, +;. gap, –. (B) Graphical view of domain composition of *Escherichia coli TmcA* and *S. pombe Nat10*. The domains are indicated as rectangles with the name indicated on the top of each rectangle. The domain name, length, and position were obtained from Pfam (http://pfam.sanger.ac.uk/). The Walker A and B motifs (blue and red bars, respectively) were assigned according to Chimnaronk *et al.* (EMBO J. 2009; 28(9): 1362–73).
(TIFF)

Figure S4 Sequence alignment of SSU rRNAs proximal to the two acetylation sites. Sequences were obtained from the EMBL database (http://www.ebi.ac.uk/) and aligned by ClustalW (http://www.clustal.org/). Asterisks indicate the fully conserved residues among the sequences. Taxonomy, accession number, and position are indicated to the left. The sequence encoding the helix is colored, and its number as defined by Yusupov *et al.* (Science. 2001; 292(5518): 883–96) is indicated on the alignment. The acetylcytidines are enclosed by rectangles.
(TIFF)

Figure S5 Secondary structure of the 3′ half of *S. pombe* 18S rRNAs. The structure taken from Silva (http://www.arb-silva.de/) is shown with the helix number defined as reported by Yusupov *et al.* (Science. 2001; 292(5518): 883-96). Ac, acetyl residue; -, hydrogen bond; •, GU mismatch.
(TIFF)

Table S1 *Schizosaccharomyces pombe* strains used in this study.
(XLSX)

Table S2 Oligonucleotides used in this study.
(XLSX)

Table S3 Post-transcriptional modifications in *Schizosaccharomyces pombe* 18S rRNA identified by LC-MS.
(XLSX)

Table S4 Sequence identity of Nat10 proteins compared with *Schizosaccharomyces pombe*.
(XLSX)
