# Supplementary figures and images for: RNA Cytidine Acetyltransferase of Small-Subunit Ribosomal RNA: Identification of Acetylation Sites and the Responsible Acetyltransferase in Fission Yeast, Schizosaccharomyces pombe
Source: PLoS One. 2014 Nov 17;9(11):e112156. doi: 10.1371/journal.pone.0112156 (PMC4234376; doi:10.1371/journal.pone.0112156)

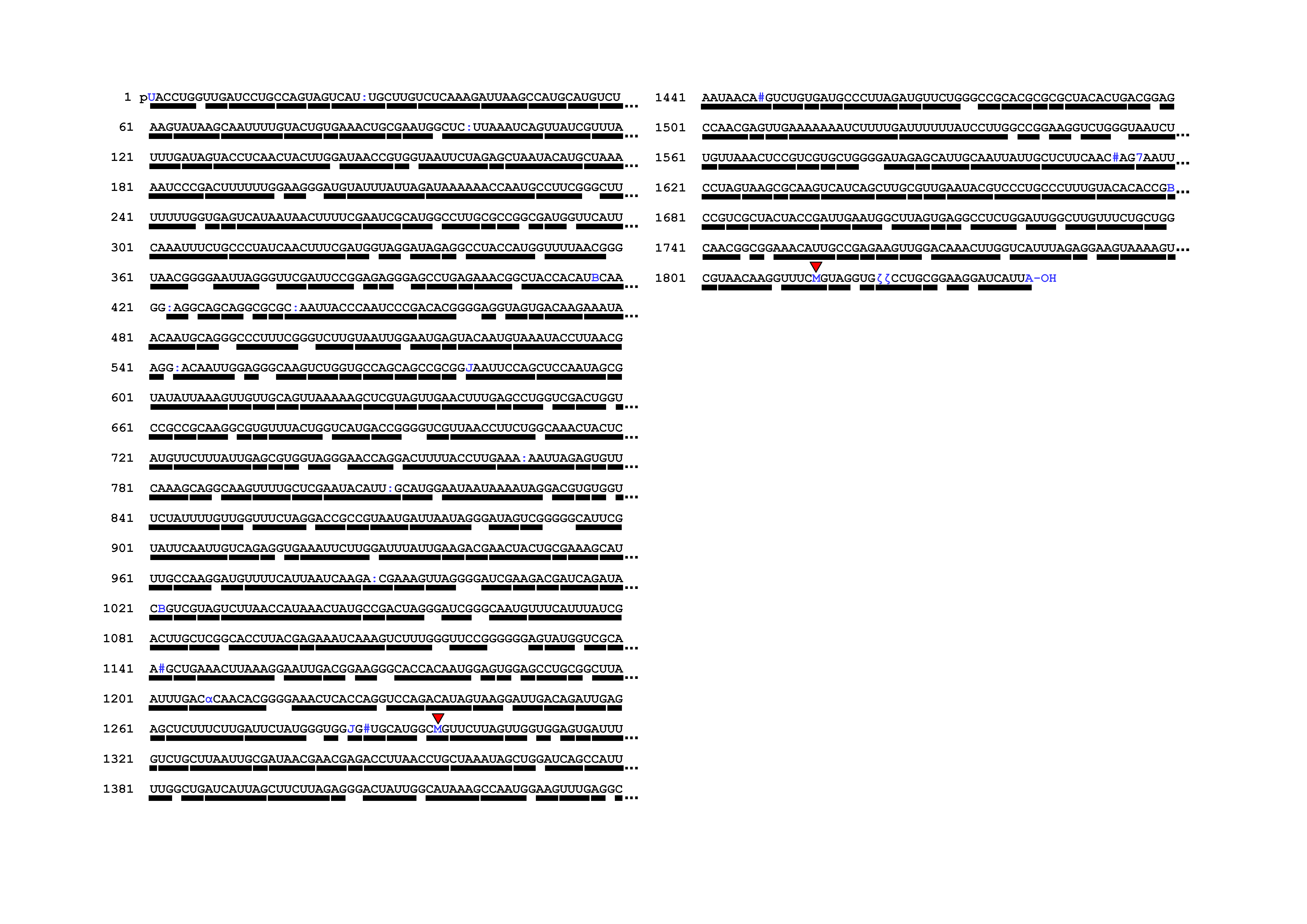

Supplement: Figure S1 — Nucleotide sequence of S. pombe 18S rRNA with the sites of modification identified by the LC-MS/MS. Black solid bars denote RNase T1–digested 18S rRNA fragments identified by this analysis. When the bar continues to the next line, a dotted line is added to the bar. The sequences with or without modifications were identified by Ariadne and confirmed by manual inspection of the MS/MS spectra. Each N-acetylcytidine is indicated by a red arrowhead. The fragment carrying modified residues is noted in Table S3. Abbreviations of modified residues (blue letters) are as follows: 2′-O-methyladenosine; B, 2′-O-methylcytidine, #, 2′-O-methylguanosine; J, 2′-O-methyluridine; M, N4-acetylcytidine; α, 1-methyl-3-(3-amino-3-carboxypropyl) pseudouridine; 7, 7-methylguanosine; ζ, N6,N6-dimethyladenosine. (TIFF) [file pone.0112156.s001.tiff]

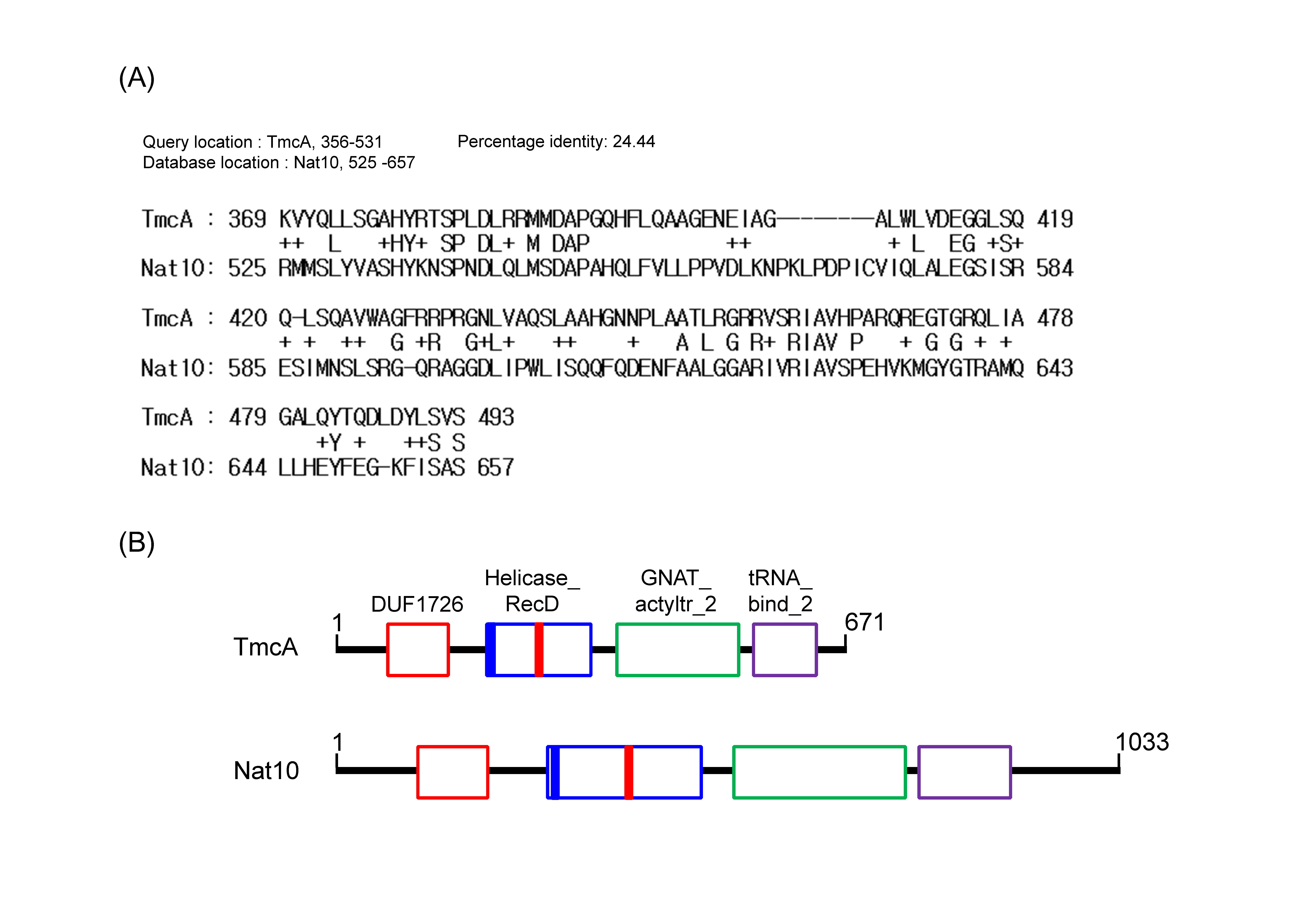

Supplement: Figure S3 — Structural similarity of Escherichia coli TmcA and S. pombe Nat10. (A) The N-acetyltransferase domain of TmcA was used to search for a homolog in S. pombe (http://www.pombase.org/). The domain shows sequence similarity with the acetyltransferase domain of Nat10. Amino acid identity: strong similarity, +;. gap, –. (B) Graphical view of domain composition of Escherichia coli TmcA and S. pombe Nat10. The domains are indicated as rectangles with the name indicated on the top of each rectangle. The domain name, length, and position were obtained from Pfam (http://pfam.sanger.ac.uk/). The Walker A and B motifs (blue and red bars, respectively) were assigned according to Chimnaronk et al. (EMBO J. 2009; 28(9): 1362–73). (TIFF) [file pone.0112156.s003.tiff]

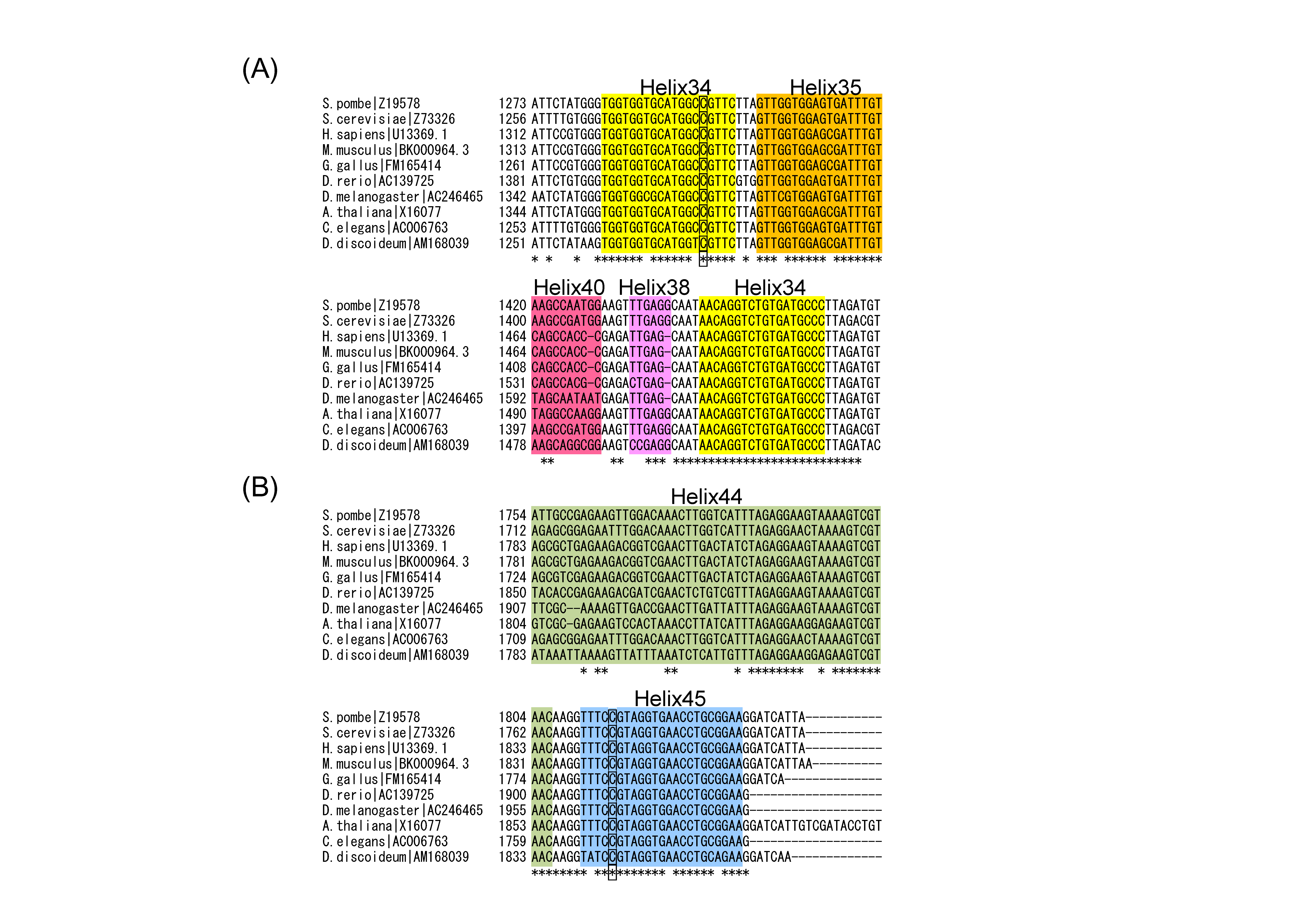

Supplement: Figure S4 — Sequence alignment of SSU rRNAs proximal to the two acetylation sites. Sequences were obtained from the EMBL database (http://www.ebi.ac.uk/) and aligned by ClustalW (http://www.clustal.org/). Asterisks indicate the fully conserved residues among the sequences. Taxonomy, accession number, and position are indicated to the left. The sequence encoding the helix is colored, and its number as defined by Yusupov et al. (Science. 2001; 292(5518): 883–96) is indicated on the alignment. The acetylcytidines are enclosed by rectangles. (TIFF) [file pone.0112156.s004.tiff]

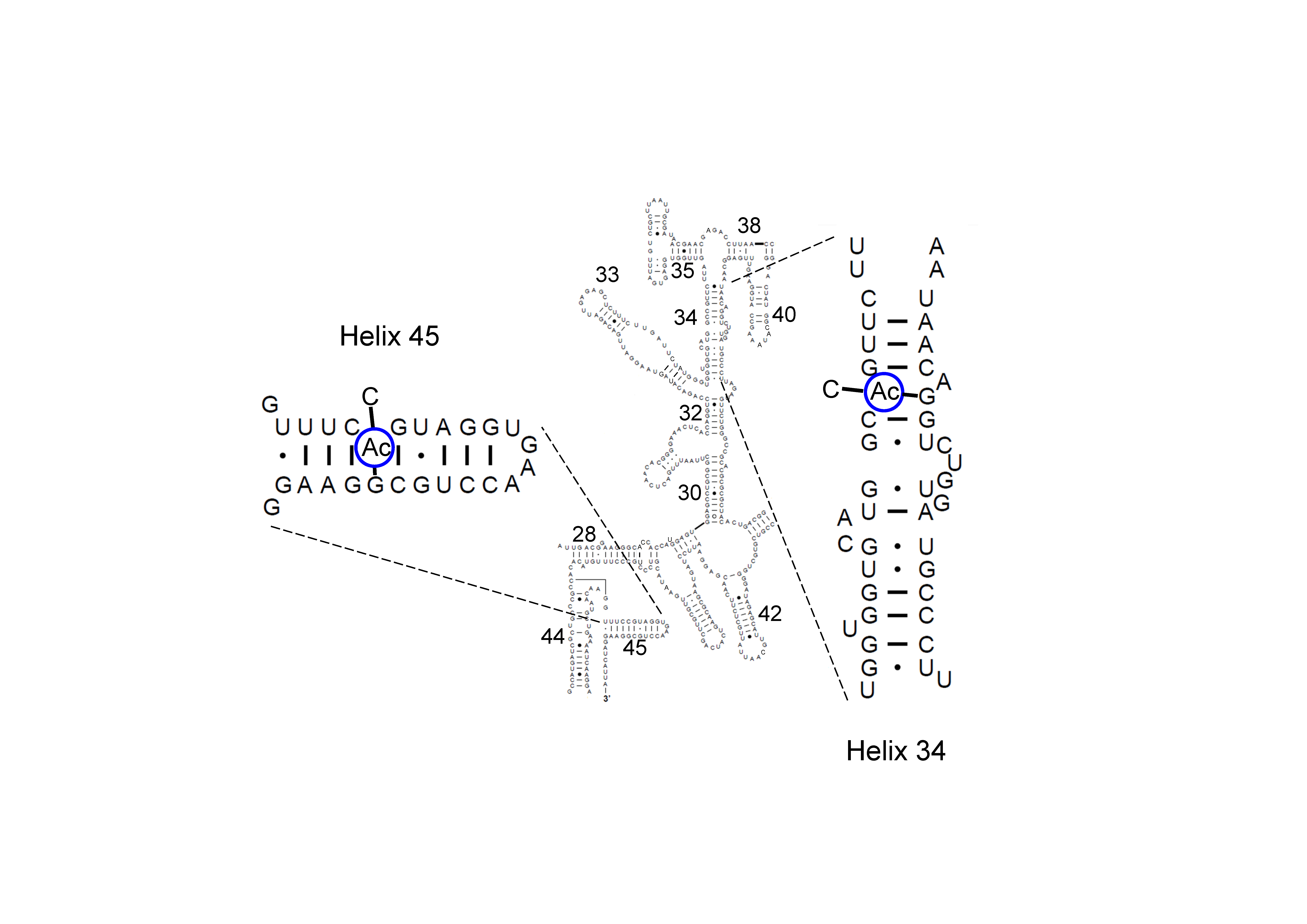

Supplement: Figure S5 — Secondary structure of the 3′ half of S. pombe 18S rRNAs. The structure taken from Silva (http://www.arb-silva.de/) is shown with the helix number defined as reported by Yusupov et al. (Science. 2001; 292(5518): 883-96). Ac, acetyl residue; -, hydrogen bond; •, GU mismatch. (TIFF) [file pone.0112156.s005.tiff]
